# Hepatotoxicity during Treatment for Tuberculosis in People Living with HIV/AIDS

**DOI:** 10.1371/journal.pone.0157725

**Published:** 2016-06-22

**Authors:** Carolline Araújo-Mariz, Edmundo Pessoa Lopes, Bartolomeu Acioli-Santos, Magda Maruza, Ulisses Ramos Montarroyos, Ricardo Arraes de Alencar Ximenes, Heloísa Ramos Lacerda, Demócrito de Barros Miranda-Filho, Maria de Fátima P. Militão de Albuquerque

**Affiliations:** 1 Departamento de Medicina Tropical, Universidade Federal de Pernambuco, Recife, Brazil; 2 Laboratório de Virologia, Centro de Pesquisas Aggeu Magalhães/FIOCRUZ, Recife, Brazil; 3 Hospital Correia Picanço, Secretaria de Saúde de Pernambuco, Recife, Brazil; 4 Instituto de Ciências Biológicas, Universidade de Pernambuco, Recife, Brazil; 5 Faculdade de Ciências Médicas, Universidade de Pernambuco, Recife, Brazil; 6 Departamento de Saúde Coletiva, Centro de Pesquisas Aggeu Magalhães/FIOCRUZ, Recife, Brazil; Public Health Agency of Barcelona, SPAIN

## Abstract

Hepatotoxicity is frequently reported as an adverse reaction during the treatment of tuberculosis. The aim of this study was to determine the incidence of hepatotoxicity and to identify predictive factors for developing hepatotoxicity after people living with HIV/AIDS (PLWHA) start treatment for tuberculosis. This was a prospective cohort study with PLWHA who were monitored during the first 60 days of tuberculosis treatment in Pernambuco, Brazil. Hepatotoxicity was considered increased levels of aminotransferase, namely those that rose to three times higher than the level before initiating tuberculosis treatment, these levels being associated with symptoms of hepatitis. We conducted a multivariate logistic regression analysis and the magnitude of the associations was expressed by the odds ratio with a confidence interval of 95%. Hepatotoxicity was observed in 53 (30.6%) of the 173 patients who started tuberculosis treatment. The final multivariate logistic regression model demonstrated that the use of fluconazole, malnutrition and the subject being classified as a phenotypically slow acetylator increased the risk of hepatotoxicity significantly. The incidence of hepatotoxicity during treatment for tuberculosis in PLWHA was high. Those classified as phenotypically slow acetylators and as malnourished should be targeted for specific care to reduce the risk of hepatotoxicity during treatment for tuberculosis. The use of fluconazole should be avoided during tuberculosis treatment in PLWHA.

## Introduction

The treatment of tuberculosis (TB) in PLWHA has been a constant challenge for medicine. The occurrence of adverse reactions and the possibility of drug interactions may interfere negatively with the therapeutic regimen, leading, in most cases, to its being discontinued [1].

Hepatotoxicity is a serious adverse reaction frequently observed during treatment for TB [2], and the incidence rate may vary between 2–28% [3]. Some studies have indicated that the occurrence of hepatotoxicity during the use of anti-tuberculosis drugs is similar in both HIV co-infected and HIV-uninfected individuals [4,5]. However, Hoffman et al. [6] found a higher incidence of hepatotoxicity among PLWHA.

In general, the risk of hepatotoxicity is influenced by genetic and acquired factors [7,3]. There is no consensus on the risk factors for developing hepatotoxicity during TB treatment. Some studies have considered the main factors that predispose towards hepatotoxicity include getting older (> 50 years), being of the female gender, using alcohol, having a low body weight, suffering from alcoholism, chronic hepatitis B and/or C, HIV infection and extra-pulmonary TB [3,8–11]. Other studies have suggested the involvement of genetic risk factors for hepatotoxicity when anti-TB drugs are used: namely, the slow acetylator phenotype of the Arylamine N-Acetyltransferase (NAT2) gene, the heterozygous c1/c2 and the homozygous c1/c1 for cytochrome (CYP) 2E1, the absence of human leukocyte antigen (HLA)-DQA1*0102 and the presence of HLA-DQB1*0201 [12–14].

In Brazil, few studies have been conducted in order to verify the relationship between the slow acetylator phenotype of the NAT2 gene and the occurrence of hepatotoxicity during TB treatment [7, 15–17]. To the best of our knowledge, no studies have been conducted exclusively with PLWHA in any of the Brazilian states, including that of Pernambuco, which records the highest incidence rate of TB in the whole of the Northeastern region of Brazil (49.3/100,000 inhabitants). In Pernambuco, a TB-HIV coinfection rate of 11.6% has been identified, which is well above that recorded for both the whole country (9.8%) and for the Northeast Region (8.1%) [18]. It is worth noting that these data may be underestimated due to the underreporting of TB/HIV coinfection, already described in Brazil [19].

Being able to characterize such parameters within the population is very useful, since the incidence and predictors of drug-induced hepatotoxicity may vary between populations, partly due to host genetics and environmental factors.

Furthermore, it is difficult to draw comparisons between the studies published due to differing definitions being used in the literature for hepatotoxicity during TB treatment. This may be corroborated by noting the findings of Coca et. al. [2] in a case-control study. This was conducted to compare the occurrence of hepatotoxicity when patients with and without HIV co-infection were being treated for TB. Coca et. al. [2] used three definitions of hepatotoxicity simultaneously and found that small changes in the definition bring about significant impacts on the results. In our study, we have chosen to use the definition adopted by the Brazilian Ministry of Health for hepatotoxicity, as applied to the country’s public health system [20]. As most studies on hepatotoxicity during TB treatment in PLWHA have been conducted in Africa [21,22] and Asia [23,24], there is a need for specific reports regarding the population in Brazil infected with HIV. This prospective cohort study has aimed to determine the incidence of hepatotoxicity and to identify predictive factors for developing hepatotoxicity after PLWHA start treatment for TB.

## Methods

The study was approved by the Committee for Ethics in Research of the Universidade Federal de Pernambuco (CEP/ CCS / UFPE 254/05), and all participants in the study signed a term of informed consent.

### Design, site and study population

This was a prospective cohort study with PLWHA of both sexes, aged ≥ 18 years, treated at the Hospital Correia Picanço (HCP), which offers a specialized service to approximately half of the PLWHA in the state of Pernambuco.

An estimate was made of the size of sample needed for this study considering the NAT2 gene polymorphism as exposure to the development of hepatotoxicity. This drew on the study by Teixeira et. al. [7], which resulted in a frequency equal to 69.2% of the "slow acetylator" NAT2 phenotype in patients who develop hepatotoxicity and an estimated odds ratio of 2.86 (95% CI: 1.06 to 7.68). Based on these parameters, using a confidence level of 95% and power of 80%, the estimated sample was 150 patients.

The individuals who started treatment for TB and were included in this study were those who provided blood samples for measuring the aspartate aminotransferase (AST) and alanine aminotransferase (ALT) enzymes before treatment for TB was started. Those who did not return for further assessment of their transaminase levels were considered lost to follow-up and those who did not present baseline ALT and/or AST measurements were excluded. Those who agreed to participate in the study signed an Informed Consent Form (ICF), based on the ethical aspects of Resolution No. 466/12 of the Brazilian National Health Council.

### Cohort follow-up

Individuals were included in the study just before TB treatment was started, at the time when compulsory notification to the Information System for Notifiable Diseases—TB (TB Sinan) was made. This is a prerequisite for initiating TB treatment in Brazil. They were monitored during the first 60 days of treatment.

Patients were interviewed by health professionals who had been previously trained how to apply a structured questionnaire. In addition, information was extracted from medical records and recorded on a data collection form. Both instruments were specifically constructed to collect data for this research.

Blood was collected from all individuals studied at baseline and on their monthly visits to the health service, during the first 60 days of TB treatment, irrespective of whether or not they presented symptoms of hepatitis after their liver enzymes had been measured. Individuals were also interviewed in order to monitor hepatitis symptoms and any adverse effects arising from TB treatment, during their first and second follow-up visits.

### Defining variables of the study

The event of interest was hepatotoxicity, defined as high aminotransferase levels and identified as being three times higher than it was before TB treatment began, with associated symptoms of hepatitis. These symptoms were considered as was the occurrence of one or more of jaundice, nausea, vomiting, dyspepsia and asthenia [20]. The reference values adopted were AST-36UI/mL and ALT-32UI/mL.

We analyzed the factors potentially associated with the development of hepatotoxicity: biological and lifestyle variables: gender, age, skin color, body mass index (BMI) which was categorized as malnourished (BMI <18.5 kg / m²), eutrophic (18.5 ≤ BMI ≥ 24.9 kg/m²) and overweight/obese (BMI ≥ 25.0 kg / m²), use of alcohol and use of illicit drugs.

To classify the patterns of alcohol consumption, information was gathered regarding the intake and frequency of alcoholic drinks over the previous three months.

The frequency of alcohol use was coded into categories ranging from “never” (for tee-totallers) to “every day” it also includes the number of units. Individuals were classified as “tee-total” (if they never drink alcohol or drink less than eight units per year); a “light drinker” (if they drink not exceeding 10 units, on a monthly basis or on not more than one or two days a week); a “heavy drinker” (if they drink in excess of five units daily or 3–4 days a week), and “alcohol dependent” (in treatment). For the purpose of the regression analysis, the variable of “alcohol consumption” was categorized into two levels: “Yes” (light drinker, heavy drinker and alcohol dependent) and “No” (tee-totallers).

Use of illicit drugs was categorized as “Current use” for individuals who reported having used marijuana, cocaine, crack and/or glue during the previous year, “Past use” for individuals who reported that they had used these drugs during their lifetime, and “Never used” for individuals who had never used these drugs.

### Clinical and laboratory variables

These variables were drug regimen for TB treatment, the use of highly active antiretroviral therapy (HAART)—(Yes/No), classes of combination antiretroviral therapy (ART), Arylamine N-Acetyltransferase (NAT2) Phenotype Gene, Serology anti-HBc (antibody to hepatitis B core antigen) and anti-HCV (hepatitis C virus antibody), and CD4 count.

The treatments for TB, according to the Brazilian Ministry of Health, were categorized and included the RIP Regimen (consisting of three drugs: rifampicin, isoniazid and pyrazinamide), the new recommended RIPE regimen (consisting of four drugs: rifampicin, isoniazid, pyrazinamide and ethambutol) and some others [20].

The use of antiretroviral drugs at the time of initiating TB treatment was confirmed by consulting medical records and categorized as: not using HAART, initiated HAART before TB treatment and initiated HAART during the first two months of TB treatment.

The use of drugs (sulfadiazine, azithromycin, sulfamethoxazole and trimethoprim, amphotericin B, fluconazole, and ganciclovir) for the treatment of other opportunistic infections during the first 60 days of TB treatment was also investigated and categorized as “Yes” for individuals who used one or more of the drugs, and “No” for individuals who used none of the drugs.

Serology for anti-HBc and anti-HCV were categorized as reactive and non-reactive. For the analysis, we considered a CD4 count measured up to six months before initiating TB treatment, and the variable was categorized as <200 cells/mm³ and ≥ 200 cells/mm^3^.

We considered individuals as seropositive for HIV, when they tested positive by two sequential tests, which is in accordance with the recommendations of the Ministry of Health in Brazil. Therefore, the result of all the 346 tests for HIV antibodies conducted on the 173 patients included in this study were positive.

We considered individuals with active TB as those who had initiated TB treatment due to clinical diagnosis or after bacteriological confirmation of TB diagnosis. All cases were reported to Sinan-TB.

### Laboratory procedures

The serological test used for detecting HIV infection was the Rapid-check HIV1 & 2 (NDI-Núcleo de Doenças Infecciosas, Vitória, ES, Brasil) or the rapid test—HIV- 1/ 2 Bio-Manguinhos (Fundação Oswaldo Cruz/ Biomanguinhos, Rio de Janeiro, Brasil) followed by a confirmatory test (western blot (WB), immunoblot (IB) or rapid immunoblot).

The CD4+ T lymphocyte count was performed by flow cytometry using anti-CD4 antibodies labeled with fluorescent dyes using the BD FACS Calibur™ system.

Serological testing for total anti-HBc and anti-HCV was conducted using the ARCHITEC i 2.000 (Abbott) analyzer, which uses the chemiluminescence method and the manufacturer's instructions were followed.

### Genotyping the NAT2 gene

NAT2 genotypes were defined by direct sequencing, comprising two *loci*. Genomic DNA was isolated using the Bio Pur Cent Mini Extraction Kit (Biometrix^®^, Brazil) in accordance with the manufacturer's specifications. After extraction, DNA samples were stored at -20°C until use. PCRs were performed with two independent reactions, as follows: 36,5μl H2O, 5μl 10x PCR buffer, 2 μl MgCl2 50 mM, 1μl 10mM dNTP, 2μl of each 10 mM primer, 1μl DNA, and also 0.5 μl Taq pol (5U/microl) to 50 μl final volume. SNPs G191A (rs1801279), C282T (rs1041983) and T341C (rs1801280) were evaluated using primers 5'-CCTTACAGGGTTCTGAGACTAC-3' and 5'-GGTGCCTTGCATTTTCTGCT-3’ and the following cycling parameters: initial denaturation (94°C/3 min), 35 cycles (94°C/1 min, 50°C/1 minute., and 72°C/1 min), and final extension at 72°C/5 minutes. On the other hand, SNPs C481T (rs1799929), G590A (rs1799930), A803G (rs1208) and G857A (rs1799931) were evaluated using primers 5'-CATTGTCGATGCTGGGTCTG-3' and 5'-TCATCCATAAAAATGTCAGCATTT-3’ with the same cycling parameters listed above, except for the annealing temperature (55°C).

The 667bp and 665bp amplicons were first analyzed by electrophoresis (1% agarose). Direct sequencing was performed using the ABI PRISM BigDye Terminator v.3.1 Sequencing Kit (PE Applied Biosystems) followed by genotyping using the ApE-A- plasmid-Editor v2.0.45 and AY331807 GenBank sequence as reference (<www.ncbi.nlm.nih.gov/nuccore/AY331807>), as recommended by the International NAT2 Gene Nomenclature Committee (<http://nat.mbg.duth.gr>).

### Phenotypic inference based on genomic data of the NAT2 gene

Among the 7 SNPs commonly found in the human population, for phenotypic classification, we analyzed four SNPs that result in amino acid substitution and the consequent decrease in the capacity of acetylation. Individuals who presented at least two variant alleles in any of the SNPs at positions 191, 341, 590 and 857 were classified as slow acetylators [25].

### Statistical Analysis

Data were stored in a database created for this research. All data were double entered (validated in EPI-INFO 6.04), and were subsequently compared to identify possible typing errors.

To check the statistical significance of differences in the frequency distribution of variables in accordance with hepatotoxicity, we used the chi-square test and, when necessary, the Fisher exact test. We carried out bivariate logistic regression analysis and the magnitude of the associations was expressed by the odds ratio (OR) with a confidence interval of 95% regression. To adjust the associations regarding possible confounding factors, we conducted a multivariate logistic regression analysis and used the statistical significance level of 20% (p <0.20) in the bivariate analysis as the criterion for entering variables into the model. The criterion for the variables that remained in the final model was their association with hepatotoxicity with a statistical significance of 5% (p <0.05). Analysis was performed using STATA 12.0 (Statistical Software for Professionals, StataCorp LP, UK).

## Results

**Fig 1:**
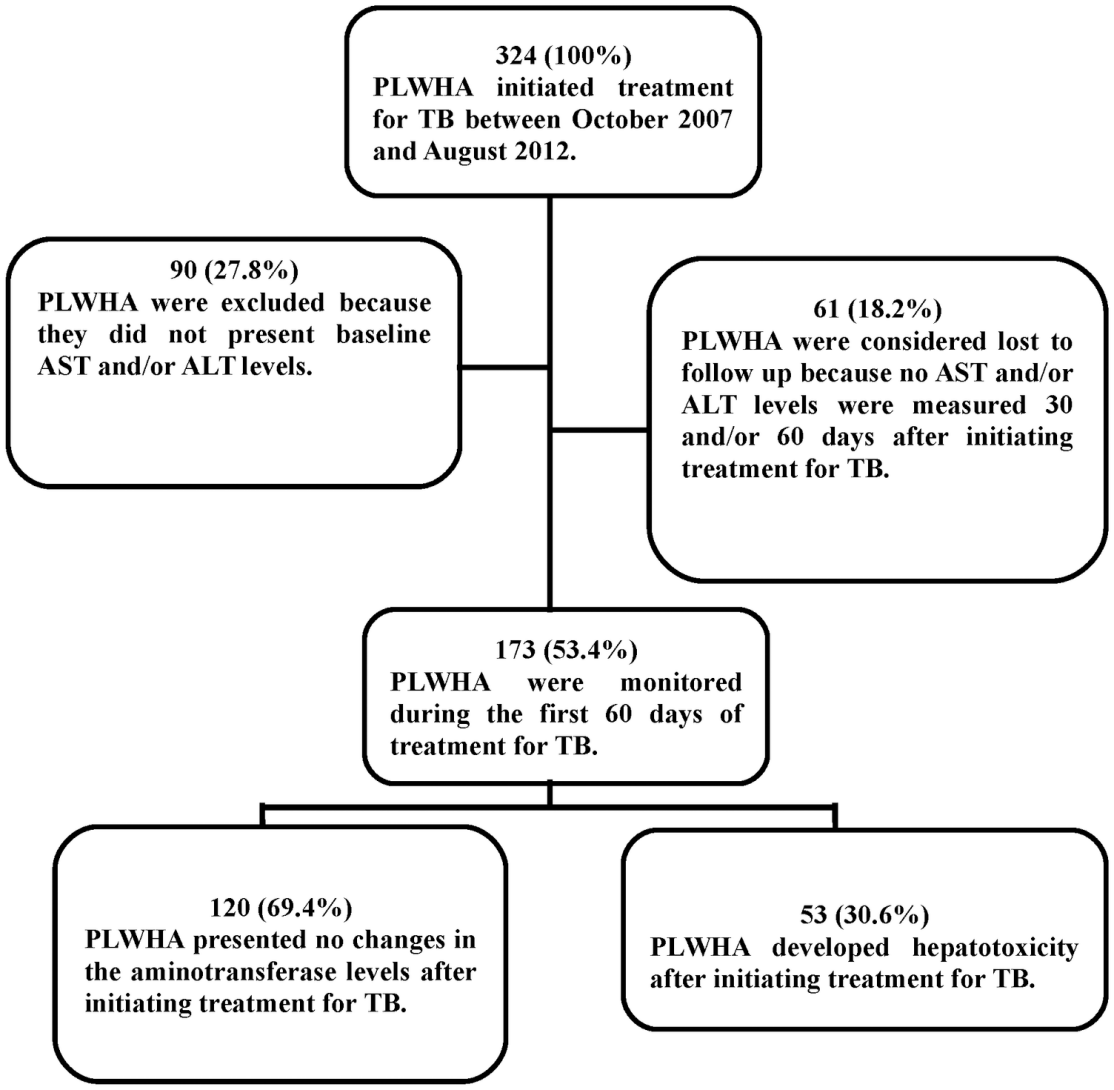
Flowchart of the study population.

Of the 324 individuals who initiated treatment for TB during the study period, 173 (53.4%) met the inclusion criteria. Of the 151 (46.6%) patients who were not included in the study, 90 (27.8%) were excluded because they did not present baseline ALT and/or AST measurements and, 61 (18.2%) were considered lost to follow up since they failed to return to the service in order to measure their AST and/or ALT levels within 30 and/or 60 days after initiating treatment for TB (Fig 1).

The study population was compared with the group of individuals who were not included in the study (151/324) with regard to sex, age, CD4 cell count and use of HAART. They were similar for all these factors except in respect of the use of HAART. The proportion of those using HAART who were lost to follow up or were excluded was 58.9% (89/151), while amongst the individuals studied this percentage was 78.6% (136/173).

**Fig 2:**
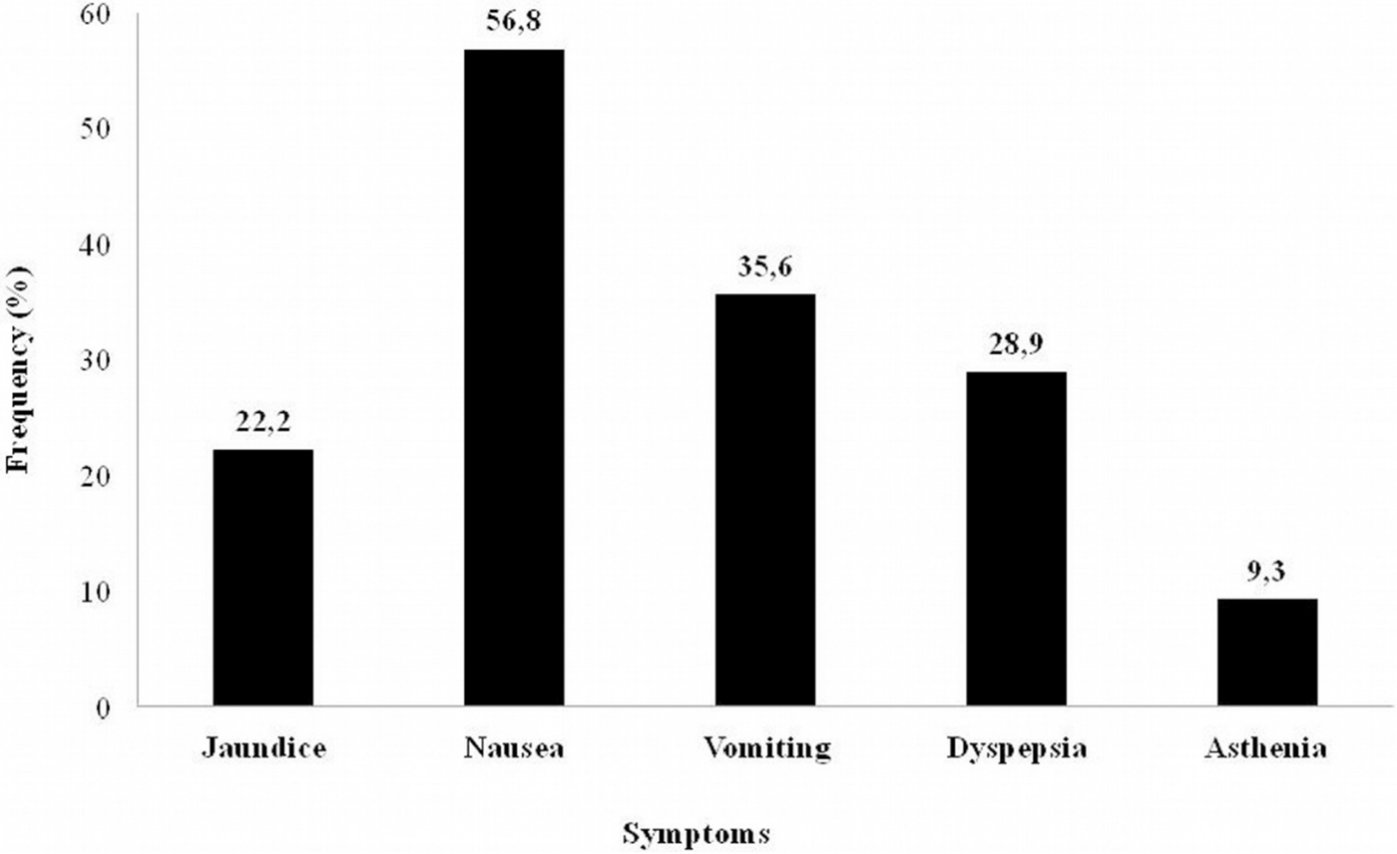
Frequency of symptoms among cases of hepatotoxicity.

Of the 173 patients studied, 53 (30.6%, 95% CI: 23.6 to 37.5) presented symptoms consistent with hepatitis (Fig 2) and high aminotransferase levels, three times higher than those recorded before TB treatment began, and these were the cases of hepatotoxicity.

Tables 1 and 2 demonstrate the frequency distribution of biological factors and lifestyle habits, clinical and laboratory factors. These tables also demonstrate the crude association between all the variables studied and hepatotoxicity.

**Table 1:** Association between biological factors and lifestyle habits and hepatotoxicity in PLWHA and TB. Recife, Pernambuco State, Brazil, 2007–2012.

	Hepatotoxicity								
Variables	Yes		No		Total		OR	CI (95%)	p-value
	N	%	N	%	N	%			
**Sex**									
Male	32	27.6	84	72.4	116	67.1	1	-	
Female	21	36.8	36	63.2	57	32.9	1.5	(0.8–3.0)	0.216
**Age (years)**									
< 40	30	33.7	59	66.3	89	51.5	1	-	
≥40	23	27.4	61	72.6	84	48.5	0.7	(0.4–1.4)	0.368
**BMI**									
Eutrophic	25	23.8	80	76.2	105	60.7	1	-	
Malnourished	22	50.0	22	50.0	44	25.4	3.2	(1.5–6.7)	0.002
Overweight/ Obese	2	11.7	15	88.3	17	9.8	0.4	(0.1–2.0)	0.279
**Skin color**									
White	14	38.9	22	61.2	36	20.8	1	-	
Non-white	39	28.5	98	71.5	137	79.2	0.6	(0.3–1.4)	0.230
**Use of Illicit drug**									
Never used	38	29.1	93	70.9	131	75.7	1	-	
Past use	13	44.8	16	55.2	29	16.7	1.9	(0.8–4.5)	0.102
Current use	2	15.4	11	84.6	13	7.5	0.4	(0.1–2.1)	0.307
**Alcohol consumption**									
No	45	31.7	97	68.3	142	82.1	1	-	
Yes	8	28.6	20	71.4	28	16.2	0.8	(0.4–2.1)	0.745

BMI (Body mass index) OR = odds ratio; CI (95%): confidence interval.

**Table 2:** Association between clinical and laboratory factors and hepatotoxicity in PLWHA and TB. Recife, Pernambuco State, Brazil, 2007–2012.

	Hepatotoxicity								
Variables	Yes		No		Total		OR	CI(95%)	p-value
	N	%	N	%	N	%			
**TB Regimen**									
RIP	35	33.1	71	66.9	106	61.3	1	-	-
RIPE	18	27.7	47	72.3	65	37.6	0.8	(0.4–1.5)	0.465
Others	0	00.0	2	100.0	2	1.2	-	-	-
**HAART use**									
Not using HAART	11	29.7	19	70.3	37	21.4	1	-	-
Initiated HAART before TB treatment	24	28.3	61	71.7	85	49.1	1.3	(0.5–3.2)	0.584
Initiated HAART during the first two months of TB treatment	18	35.3	33	64.7	51	29.5	0.9	(0.4–2.2)	0.867
**Classes of combination ARV**									
Not using ARV	11	29.7	26	70.3	37	21.4	1	-	-
2NRTI + 1NNRTI	31	29.8	73	70.2	104	60.1	1.0	(0.4–2.3)	0.993
2NRTI + 1PI/r	9	37.5	15	62.5	24	13.9	1.4	(0.5–4.2)	0.528
Others	2	25.0	6	75.0	8	4.6	0.8	(0.1–4.5)	0.789
**NAT2 Phenotype Gene**									
Fast acetylator	30	25.0	90	75.0	120	69.4	1	-	
Slow acetylator	23	43.4	30	56.6	53	30.6	2.3	(1.2–4.5)	0.017
**Anti-HBc**									
Non-reactive	38	36.2	67	63.8	105	60.3	1	-	
Reactive	14	22.9	47	77.1	61	36.7	0.5	(0.3–1.1)	0.078
**Anti-HCV**									
Non-reactive	49	31.6	106	68.4	155	89.6	1	-	
Reactive	3	27.3	8	72.3	11	6.4	0.8	(0.2–3.2)	0.765
**CD4 count (cels/mm³)**									
≥ 200	12	18.7	52	81.3	64	41.1	1	-	
< 200	33	35.8	59	64.2	92	58.9	2.4	(1.2–5.2)	0.022

TB (Tuberculosis), RIP (Rifampicin, Isoniazid, Pyrazinamide), RIPE (Rifampicin, Isoniazid, Pyrazinamide, ethambutol), HAART (Highly active antirretroviral therapy) ARV (Antiretroviral therapy), NRTI (nucleoside reverse transcriptase inhibitor), NNRTI (Non-nucleoside reverse transcriptase inhibitor), PI/r (Protease inhibitor/ritonavir), NAT2 (Arylamine N-Acetyltransferase), Anti-HBc (hepatitis B core antibody and antigen) and Anti-HCV(hepatitis C virus antibody).

OR (odds ratio); CI (95%): confidence interval.

The population was characterized as being predominantly male (67.1%), just over half (51.5%) were aged between 20 and 39 years, with a greater proportion of eutrophic (60.7%) and non-white (79.2%) individuals. The use of illicit drugs was reported by 7.5% of the patients and the use of alcohol by 16.2% (Table 1).

**Fig 3:**
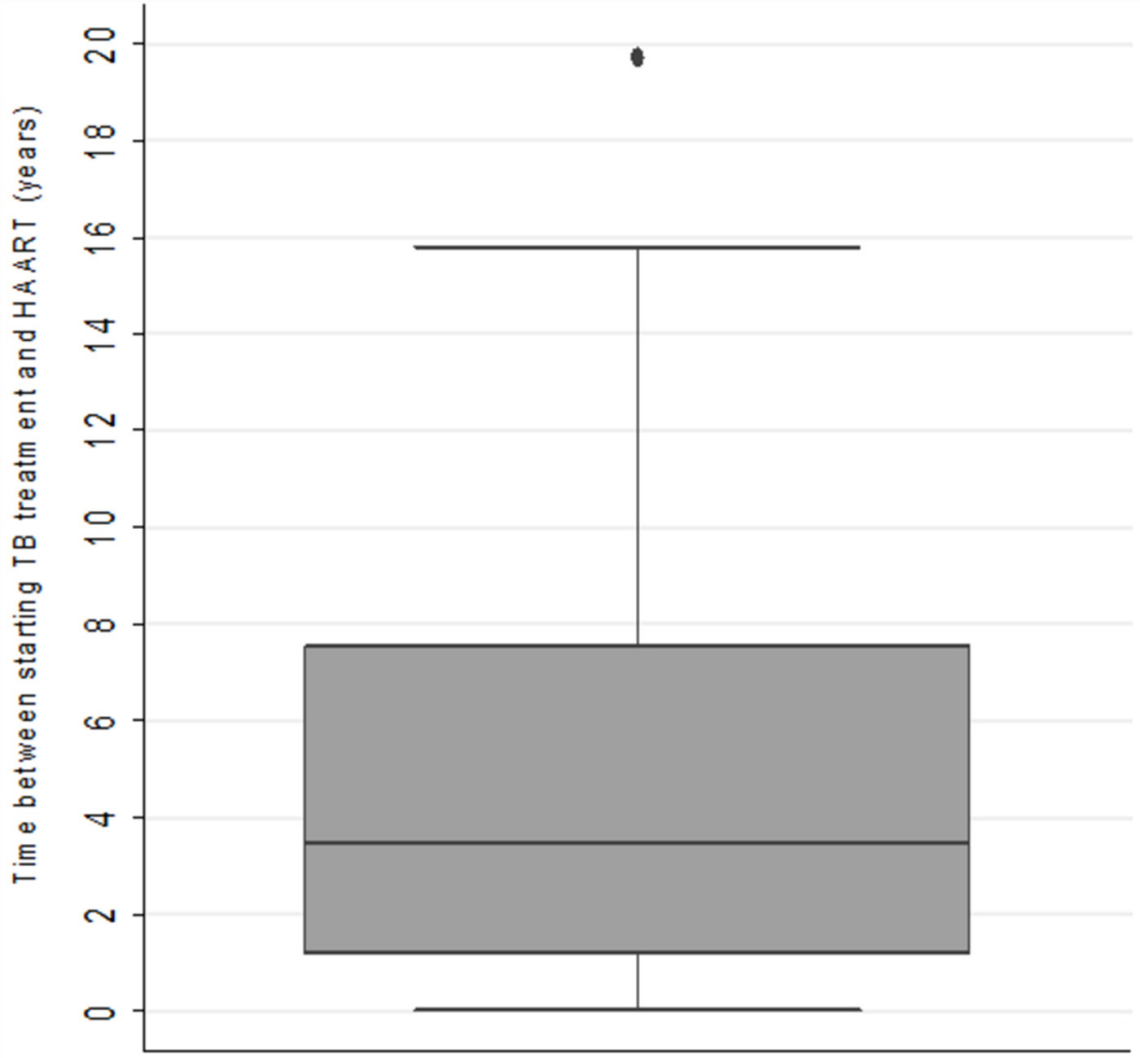
Average length of time that patients had been taking HAART regarding those who had begun ARV treatment before TB treatment.

It was found that the most commonly used therapeutic regimen for TB (61.3%) was that of three drugs (RIP), and HAART was used by 78.6% (136/173), the majority of whom had initiated HAART before treatment for TB. Of the 49.1% (85/173) who were already using HAART, the average length of time that ARV had been used was 4.7 years (SD 4.3 years) (Table 2 and Fig 3).

The antiretroviral regimen containing two NRTIs (nucleoside reverse transcriptase inhibitors) and one NNRTI (non-nucleoside reverse transcriptase inhibitor) was the one most commonly used by HAART users (60.1%). Of the 173 PLWHA evaluated for the presence of hepatotoxicity prior to beginning treatment for tuberculosis, 166 (95.9%) were investigated for the presence of HBV and HCV serological markers. Of these 166 patients studied, 61 (36.7%) were anti-HBc positive, suggesting prior contact with the virus, and 11 (6.4%) were positive for HCV (Table 2).

There was a predominance of individuals phenotypically classified as fast acetylators for NAT2 (69.4%). The prevalence of different NAT2 subtypes found is presented in Table 3. According to the International NAT2 Gene Nomenclature Committee, no new genotypic subtypes have been found for the 7 *loci* studied in the NAT2 gene.

**Table 3:** NAT2 genotypes and acetylation profile in PLWHA/TB patients according to the International NAT2 Gene Nomenclature Committee. Recife, Pernambuco State, Brazil, 2007–2012.

NAT2 Genotype	Frequency		Total	
	N	%	N	%
**Fast acetylator phenotype**			120	69.4
NAT2*4	102	85.0		
NAT2*11A	1	0.8		
NAT2*12ª	4	3.3		
NAT2*12C	6	5.0		
NAT2*13ª	7	5.8		
**Slow acetylator phenotype**			53	30.6
NAT2*5ª	2	3.7		
NAT2*5B	21	39.6		
NAT2*5C	4	7.5		
NAT2*5D	14	26.4		
NAT2*5J	1	1.8		
NAT2*6ª	9	16.9		
NAT2*6B	1	1.8		
NAT2*7B	1	1.8		

NAT2 (Arylamine N-Acetyltransferase).

The use of drugs for treating other opportunistic diseases is described in Table 4. Fluconazole and trimethoprim-sulfamethoxazole were used by 35.3% and 50.8% of subjects, respectively.

**Table 4:** Association between the drugs used to treat other opportunistic diseases and hepatotoxicity in PLWHA and TB. Recife, Pernambuco State, Brazil, 2007–2012.

	Hepatotoxicity								
Variables	Yes		No		Total		OR	CI(95%)	p-value
	N	%	N	%	N	%			
**Sulfadiazine**									
No	42	27.8	109	72.2	151	87.3	1	-	
Yes	11	50.0	11	50.0	22	12.7	2.6	(1.1–6.4)	0.040
**Azithromycin**									
No	45	30.2	104	69.8	149	86.2	1	-	
Yes	8	33.3	16	66.7	24	13.8	1.2	(0.5–2.9)	0.758
**Sulfamethoxazole and trimethoprim**									
No	23	27.1	62	72.9	85	49.2	1	-	
Yes	30	34.1	58	65.9	88	50.8	1.4	(0.7–2.7)	0.317
**Amphotericin B**									
No	51	30.5	116	69.5	167	96.5	1	-	
Yes	2	33.3	4	66.7	6	3.5	1.1	(0.2–6.4)	0.884
**Ganciclovir**									
No	50	29.6	119	70.4	169	97.7	1	-	
Yes	3	75.0	1	25.0	4	2.3	7.2	(0.7–70.3)	0.092
**Fluconazole**									
No	24	21.4	88	78.6	112	64.7	1	-	
Yes	29	47.5	32	52.5	61	35.3	3.3	(1.7–6.5)	<0.001

OR (Odds ratio), CI (95%) (Confidence interval)

The final multivariate logistic regression model for the factors associated with hepatotoxicity during the first 60 days of TB treatment is presented in Table 5. The variables that remained in the model, thus increasing the chance of hepatotoxicity were the use of fluconazole, malnutrition (BMI <18.5) and being phenotypically classified as NAT2 slow acetylators.

**Table 5:** Multivariate model of the association between the factors studied and hepatotoxicity in PLWHA and TB. Recife, Pernambuco State, Brazil, 2007–2012.

Variables	Odds Ratio	CI (95%)	P-value
**NAT2 Phenotype Gene**			
Fast acetylator	1	-	
Slow acetylator	2.1	(1.0–4.3)	0.053
**BMI**			
Eutrophic	1	-	
Malnourished	3.0	(1.4–6.6)	0.006
Overweight/ Obese	0.6	(0.1–2.7)	0.472
**Fluconazole**			
No	1	-	
Yes	3.0	(1.5–6.2)	0.002

NAT2 (Arylamine N-Acetyltransferase), BMI (Body mass index)OR (odds ratio); CI (95%): confidence interval.

## Discussion

The cumulative incidence rate of hepatotoxicity in PLWHA following the use of drugs for TB treatment was 30.6% in the population studied. The use of fluconazole, being malnourished and a NAT2 slow acetylator phenotypic profile were factors independently associated with hepatotoxicity.

This rate of hepatotoxicity observed in our study was very similar (30%) to the rate reported in a study conducted in Ethiopia [21]. Another study, conducted in Brazil, demonstrated a higher rate of hepatotoxicity (43.8%) amongst those infected with HIV and a rate of 30.8% in seronegative HIV patients [26]. However, the comparison of our results with other studies presents limitations, since, as mentioned above, there are differing definitions of hepatotoxicity found in the literature which hinders comparisons between published studies. The studies cited above [8,14], unlike the present study, adopted the classification criteria of the Council for International Organizations of Medical Science (CIOMS) which defines hepatotoxicity as high ALT levels which are twice the upper limit of normal [27]. However, it is very useful to choose the criterion of hepatotoxicity recommended by the Ministry of Health of Brazil for individuals taking antituberculosis drugs, given that this definition provides guidelines for clinical decision-making when managing sick individuals.

In PLWHA, TB treatment follows the same recommendations as those given for the general population, both in relation to therapeutic regimens and to the total duration of treatment. Nevertheless, the treatment of TB in PLWHA is a complex problem because it involves the question of identifying the toxicity of the two therapeutic regimens, the requirements of medication adherence and the inherent problems regarding the association between the two diseases. Thus, the simultaneous initiation of both treatments, for HIV infection and for TB, besides presenting an increased risk of drug intolerance and causing imposing the difficulty of identifying which drug is linked to a possible toxicity, increases the risk of adverse reactions [20,28].

In our study population, individuals phenotypically classified as slow acetylators presented a higher chance of developing hepatotoxicity than those classified as fast acetylators with an OR = 2.1 and a borderline p-value (p = 0.053). We kept the NAT2 phenotype in the final multivariate model because it has a borderline statistical significance and there is a biological plausibility that slow acetylators are associated with hepatotoxicity. However, this remains controversial. Mitchell et al. [29] argued that fast acetylators would be more vulnerable to liver damage induced by antituberculosis drugs due to an increased production of hepatotoxin, because of fast NAT2 enzyme activity, and Vuilleumier et. al. [30] found no relationship between the profiles of acetylation and hepatotoxicity induced by these drugs. We believe that individuals phenotypically classified as slow acetylators are more susceptible to developing hepatotoxicity, because they accumulate more acetylhydrazine, which competes directly with toxic metabolites of isoniazid by acetylation [12]. Moreover, a recent meta-analysis [31] has demonstrated an association between the NAT2 slow acetylator phenotype gene and the development of hepatotoxicity. This is in agreement with our findings. Our results are consistent with the latest published evidence in Brazilian studies and the international literature on the increased risk of hepatotoxicity in individuals phenotypically classified as slow acetylators during treatment for tuberculosis [7, 12, 15–17, 32].

We have verified that the use of fluconazole was strongly associated with the development of hepatotoxicity when fluconazole is taken during the first 60 days of treatment for tuberculosis. A retrospective study in PLWHA conducted by Pukenyte et. al. [33], in France, demonstrated similar results. This evidence suggests that the concomitant use of fluconazole with antituberculosis drugs for PLWHA increases the risk of developing hepatotoxicity. The use of antifungal drugs, including fluconazole, was reported as being associated with hepatotoxicity especially in those with other risk factors for liver injury [34], as observed in our study. Our findings indicate the need to contraindicate the use of this drug in PLWHA who are due to initiate treatment for TB, as the use of fluconazole is often prescribed to this population as a prophylactic measure to prevent the recurrence of cryptococcal disease and esophageal and oropharyngeal candidiasis, especially among patients with low CD4 cell counts.

Protein-energy malnutrition among PLWHA has been extensively studied, especially before the introduction of HAART, when weight loss and malnutrition were frequent. At the time, wasting syndrome was a relatively common complication in the advanced stages of HIV infection and responsible for 80% of mortality in AIDS patients [35–37]. With the advent of HAART, while malnutrition is still reported in PLWHA, it is less frequent [38]. The nutritional profile of the study population consisted predominantly composed of eutrophic individuals (60.7%), while 25.4% were classified as malnourished and 9.8% as overweight/obese. It was demonstrated that malnourished individuals were three times more likely to develop hepatotoxicity than eutrophic individuals. This fact may be explained by the depletion of glutathione deposits caused by the presence of malnutrition, which leads to the slowing down of the hepatic metabolism of drugs and a greater vulnerability to oxidative injury. Moreover, the literature reports that a low body mass index and hypoalbuminemia, i.e., malnutrition, are associated with high rates of hepatotoxicity induced by antituberculosis drugs [8,39,40].

One unexpected finding was that the consumption of alcohol was not associated with hepatotoxicity in the population studied. It is probable that the recommendation was made to people living with HIV/AIDS to refrain from drinking alcohol either when the viral infection was first diagnosed or before initiating HAART. In the population studied, only 16.2% reported that they drank alcohol.

A peculiar feature of this cohort was the inclusion of individuals who use different schemes for TB treatment. This was because the Ministry of Health of Brazil included in its recommendations, the use of a 4th drug, ethambutol, in the basic scheme for treating TB. This change came into force during the follow-up period of this cohort and we therefore decided to analyze whether the two groups of individuals using these different schemes presented different risks for developing hepatotoxicity. In this study, the majority (106/173) of patients took three drugs, but some (65/173) had been using the new scheme as proposed by the Brazilian Ministry of Health, since 2009. Therefore, two patients were treated with different schemes, one because a previous therapy had failed and the other because of intolerance to pyrazinamide. Nevertheless, an association between hepatotoxicity and the use of three or four drugs was not observed, as shown in Table 2.

The great majority of the study population (78.6%) used HAART, of whom 30.8% presented hepatotoxicity during the first 60 days of TB treatment. In theory, the use of HAART is associated with a higher risk of hepatotoxicity because it potentiates the hepatotoxicity effect of the anti-TB drugs. Thus, the fact that the study included more individuals on HAART when compared to individuals who were not included in the study, could have led to overestimating the existing risk, and not to reducing it. In fact, the use of HAART was not associated with hepatotoxicity, which makes the selection bias less likely.

In our study, all patients who had taken antiretroviral treatment with NNRTI used Efavirenz, with the exception of two patients who took Nevirapine. One of these patients presented with hepatotoxicity. We chose to analyse the combination of ARV classes due to the wide variation in the antiretroviral drugs used by the population studied. Certainly, if we had analysed the association of each drug with the outcome, the power of the study would be limited.

Our study has the limitations of an observational study with PLWHA attending a routine medical care setting. We did not achieve a sample size with the necessary power to demonstrate a statistically significant association between the NAT2 slow acetylator profile and the development of hepatotoxicity in PLWHA undergoing treatment for TB. However, the association found (OR = 2.1; p value = 0.053) indicates the chance of hepatotoxicity is increased among individuals with an NAT2 slow acetylator profile.

Another limitation was that recall bias may have occurred in the responses regarding the consumption of alcohol and use of illicit drugs. However, this bias may have been minimized since the questions were constructed at a level of detail that allowed the answers obtained to be regarded as sufficiently complete and reliable. Finally, we studied the exposures related to drugs registered by the attending physician. We did not investigate the concomitant use of medicinal herb in this population.

Bacteriological confirmation of tuberculosis in PLWHA is not frequent because this form of TB is paucibacillary and sputum smear microscopy has low sensitivity in this population. In addition, it takes a relatively long time to receive culture results and therefore attending physicians often initiate TB treatment without bacteriological confirmation. This absence of bacteriological confirmation may have had a low impact because this is a common situation. Therefore, in our study, 47 patients were diagnosed according to clinical criteria only. Among these, there was just one case (1/47), in which the diagnosis was changed and that patient was excluded from the sample studied.

Our findings are relevant for improving support to PLWHA in the specialized health services for HIV/AIDS where polypharmacy is very frequent. Identifying risk factors for hepatotoxicity in PLWHA who are undergoing treatment for tuberculosis may contribute to the exercise of greater control in managing drugs used by this population, especially those cases referred for the use of antifungal drugs.

Given our findings, we suggest that the use of fluconazole should be contraindicated during the first 60 days of TB treatment in PLWHA. Furthermore, we emphasize the importance of including a nutritionist on the staff of referral services for PLWHA. This would enable preventive actions to be implemented and the nutrition of patients to be monitored. We also believe that in the near future, the treatment of tuberculosis should become individualized, namely, drugs and dosages should be adapted according to the NAT2 phenotypic profile of each patient.
